# The Influence of Nanoparticles on Fire Retardancy of Pedunculate Oak Wood

**DOI:** 10.3390/nano11123405

**Published:** 2021-12-16

**Authors:** Danica Kačíková, Ivan Kubovský, Adriana Eštoková, František Kačík, Elena Kmeťová, Ján Kováč, Jaroslav Ďurkovič

**Affiliations:** 1 Faculty of Wood Sciences and Technology, Technical University in Zvolen, T.G. Masaryka 24, 96001 Zvolen, Slovakia; kacikova@tuzvo.sk (D.K.); kacik@tuzvo.sk (F.K.); xkmetovae@is.tuzvo.sk (E.K.); 2 Faculty of Civil Engineering, Technical University of Košice, Vysokoškolská 4, 04200 Košice, Slovakia; adriana.estokova@tuke.sk; 3 Faculty of Forestry, Technical University in Zvolen, T.G. Masaryka 24, 96001 Zvolen, Slovakia; kovacj@tuzvo.sk (J.K.); jaroslav.durkovic@tuzvo.sk (J.Ď.); 4 Faculty of Natural Sciences, Comenius University in Bratislava, Ilkovičova 6, 84215 Bratislava, Slovakia

**Keywords:** *Quercus robur*, nanoparticles, water glass, thermal analysis, flame retardants

## Abstract

Traditional flame retardants often contain halogens and produce toxic gases when burned. Hence, in this study, low-cost, environmentally friendly compounds that act as fire retardants are investigated. These materials often contain nanoparticles, from which TiO_2_ and SiO_2_ are the most promising. In this work, pedunculate oak wood specimens were modified with sodium silicate (Na_2_SiO_3_, i.e., water glass) and TiO_2_, SiO_2_, and ZnO nanoparticles using the vacuum-pressure technique. Changes in the samples and fire characteristics of modified wood were studied via thermal analysis (TA), infrared spectroscopy (FTIR), and scanning electron microscopy, coupled with energy-dispersive X-ray spectroscopy (SEM-EDX). The results of TA showed the most significant wood decomposition at a temperature of 350 °C, with a non-significant influence of the nanoparticles. A dominant effect of sodium silicate was observed in the main weight-loss step, resulting in a drop in decomposition temperature within the temperature range of 36–44 °C. More intensive decomposition of wood treated with water glass and nanoparticles led to a faster release of non-combustible gases, which slowed down the combustion process. The results demonstrated that wood modifications using sodium silicate and nanoparticle systems have potentially enhanced flame retardant properties.

## 1. Introduction

Wood is a natural material widely used for the construction of buildings and for the production of various building elements, furniture, and goods. It is also ecological, comfortable, and aesthetically pleasing; however, its use in building construction is limited and strictly regulated by fire and environmental safety rules because it is highly flammable. Therefore, to obtain fire-safe wood structures, fireproof materials need to be used. The fire protection agents applied to wooden structures contain ammonium phosphates or sulfates, chlorides, oxides, borates and other metal salts, boric acid, and halogen-containing flame retardants [1,2]. Many of these chemicals and their combustion products are highly toxic; therefore, new flame retardants are being sought out. Promising retarders from both environmental and economic points of view include nanomaterials [1,3].

Various nanomaterials with different application methods have been used to improve the fire resistance of wood. In the past, aluminium, boron, and halogens (e.g., bromine) and, more recently, phosphorus and nitrogen have been shown to be effective fire retardants in wood. Upon combustion, the halogenated compounds release toxic and/or highly corrosive gases, which are harmful to both humans and the environment. Halogenated flame retardants are therefore being phased out and replaced with halogen-free alternatives [4,5,6,7,8].

Nanocomposites constitute a new development in fire retardancy [9]. With the advent of nanotechnology in the past few decades, the prospects of nano scale fillers in polymer-based composites within flame retardancy applications have progressed rapidly. Although nanofillers do not inherently show excellent fire retardance, the incorporation of a low amount in polymer composites tends to provide drastic improvements in the thermal stability, smoke release amount, peak heat release rate, and speed at which flames spread throughout the nanocomposites. However, their efficiency is still insufficient in providing adequate fire retardancy when used alone in coatings. Therefore, their combination with other conventional fire retardant systems can give superior properties to the substrate. Nanocomposite-based coatings have been reported as a promising system that protects the oxidation of a char structure, once formed, and thus reinforces the fire-resistant properties of coatings [5,10,11,12]. Nanoparticles can form a coating on the surface of a material to inhibit the release of combustible gas and smoke, isolate oxygen from outside, and to prevent heat transfer. The combustible gas concentration can be diluted by the non-combustible gas produced by pyrolysis of the nanoparticles; the combustion chain reaction is suppressed by highly reactive free radicals produced by pyrolysis of the nanoparticles [13,14].

Zinc oxide nanoparticles have attracted the attention of researchers due to properties such as their biocompatibility, good chemical stability, and high absorption performance; in addition, they can serve as a heat-protective barrier for pine wood samples treated with the water suspension of ZnO [15]. Similarly, nano ZnO particles in the dispersion of potassium methyl siliconate shows a good level of fire retardancy comparable with a commercial flame retardant [16]. Rao et al. [17] found that a small amount of zinc oxide nanoparticles significantly increased the limited oxygen index values of the intumescent flame-retardant coating of plywood. The addition of ZnO nanoparticles could change the thermal degradation behaviours of coatings with increasing char residue percentages at high temperatures [17]. The impregnation of wood with Al_2_O_3_ and SiO_2_ leads to an increase in the char formation and lower thermal conductivity of the surface, as shown in the results from thermal analyses [18].

When comparing the effects of an aqueous dispersion of SiO_2_, TiO_2_, and ZrO_2_, SiO_2_ dispersion was the most effective in improving the fire properties of pine veneers [19]. In a study by Li et al. [20], titanium dioxide in association with a conventional intumescent flame retardant system that contains ammonium polyphosphate/pentaerythritol/melamine (APP–PER–MEL) was introduced to silicone-acrylate coatings. From the results obtained, its significant effect on char formation and the reduction in the spread of flames on a plywood plate were evident.

Garskaite et al. [21] reported the reinforcement of sapwood of Scots pine using aqueous formulations of sodium metasilicate and nano-TiO_2_ via a vacuum-pressure technique. Their results showed that the fixation of nano-TiO_2_ on the wood surface using an aqueous sodium silicate solution has potential in modeling low-cost and less fire-hazardous materials. Recently, Taghiyari et al. [22] found significant improvements in the fire properties of silver fir wood modified with nano-sepiolite. Erceg et al. [23] proposed two procedures for the synthesis of calcium phosphate composites with TiO_2_ nanoplates and nanowires with great potential for biomedical application; however, fire retardancy may be another area of their application. The hydrothermal method was used to modify a wood surface due to the deposition of TiO_2_/ZnO coatings at a relatively low temperature, and, as a result, the treated wood had an improved fire resistance, and the one-pot hydrothermal method was a feasible method used to fabricate non-flammable wood materials [24].

The growth of anatase TiO_2_ coating on a wood surface through the hydrolysis of tetrabutyl orthotitanate (TBOT) in different conditions, using a controlled hydrothermal method at low temperatures was reported [25]. The TiO_2_ coating effectively acts as a protective layer to prolong the duration of wood combustion and prevents harmful gases from spreading [26].

In recent years, nanoparticles such as titanium dioxide, silicon dioxide, and zinc oxide in intumescent flame retardant coatings have attracted much interest. Despite the progress made in research, many issues regarding the combination of retardants, the concentrations of substances used, methods of application, etc. are still unresolved. In addition, a comparison of the effect of these nanoparticles on wood flame retardancy under the same conditions is lacking.

The aim of this work was to assess three types of nanoparticles and sodium silicate to improve the fire resistance of oak wood.

## 2. Materials and Methods

### 2.1. Wood Treatment

Pedunculate oak (*Quercus robur* L.) specimens with dimensions of 10 × 40 × 50 mm (tangential (T) × radial (R) × longitudinal (L)) were cut from a stem harvested in central Slovakia. The specimens were conditioned in a climate chamber (20 ± 2 °C, 65 ± 3% relative humidity (RH)) for 21 days. The specimens were weighed before and after the conditioning, and the moisture content (MC) of these samples was estimated to be approximately 12% based on dry weight. The samples were divided into eight groups (untreated control; treated with 20% aqueous solution of sodium silicate (water glass, WG); treated with 3% dispersion of nanoparticles—TiO_2_, SiO_2_, and ZnO in water; and treated with 3% dispersion of nanoparticles—TiO_2_, SiO_2_, and ZnO in 20% aqueous solution of water glass (WG)). The nanoparticles were provided by Merck (Darmstadt, Germany): TiO_2_, purity ≥ 99.5%, size 21 nm; SiO_2_, purity ≥ 99.5%, size 5–15 nm; and ZnO, purity ≥ 97.0%, size < 100 nm.

Solutions of nanoparticles at a concentration of 3% were prepared for the impregnation procedure. For this purpose, the required weight of nanoparticles was dispersed in distilled water/20% solution of water glass using an ultrasonic dispenser. The wood samples were treated using the vacuum-pressure process; first, the specimens were kept in a vacuum (–5 kPa) for 2 h and then for 1 h at the pressure of 800 kPa. Afterwards, the treated samples were dried at room temperature to constant weight and then conditioned in a climate chamber (20 ± 2 °C, 65 ± 3% RH) for 7 days. The reference and modified samples were then mechanically disintegrated and milled to particle sizes of 200–300 μm using a POLYMIX PX-MFC 90D laboratory mill (Kinematica, Malters, Switzerland) and dried (4 h at 103 ± 2 °C).

### 2.2. Samples Analyses

#### 2.2.1. Thermal Analysis

Thermal analysis (TG—thermogravimetry, DTG—differential thermogravimetry, and DSC—differential scanning calorimetry) was performed on the powder wood samples using a STA F3 Jupiter thermal analyzer (Netzsch, Selb, Germany) in the temperature interval from 25 to 600 °C. The measurements were carried out under nitrogen atmosphere for wood samples with weights of 12.5 ± 0.1 mg in corundum (Al_2_O_3_) crucibles. The heating rate applied was 10 °C/min for all measurements.

#### 2.2.2. Infrared Spectroscopy

ATR-FTIR spectra of the homogeneously mixed wood powders of the untreated and modified samples were recorded using the Nicolet iS10 FT-IR spectrometer (Thermo Fisher Scientific, Waltham, MA, USA), equipped with Smart iTR using an attenuated total reflectance (ATR) sampling accessory attached to a diamond crystal. The spectra were collected in 32 scans at 4 cm^−1^ resolution over the range of 4000 to 650 cm^−1^. The data obtained were analyzed using OMNIC 9.0 software. Four replicates per sample were performed.

#### 2.2.3. Scanning Electron Microscopy–X-ray Spectroscopy Observations

The wood sections were mounted on specimen stubs, sputter-coated with gold (layer thickness of 150 nm) in the Sputter Coater K650X (Quorum Technologies, Ashford, UK) in an argon atmosphere, and examined using high-vacuum scanning electron microscopy coupled with energy-dispersive X-ray spectroscopy (SEM-EDX). SEM-EDX observations of the earlywood vessels were performed using a JEOL JSM-6390LV instrument (JEOL, Tokyo, Japan), operating at 20 kV and a working distance of 15 mm, equipped with an EDX spectroscope INCAx-act (Oxford Instruments, Abingdon, UK). The elemental composition of the nanoparticles and water glass was assessed on the radial and tangential cell wall surfaces of three specimens per treatment, with 3–6 measurements per specimen.

## 3. Results and Discussion

### 3.1. Thermal Analysis

**Figure 1:**
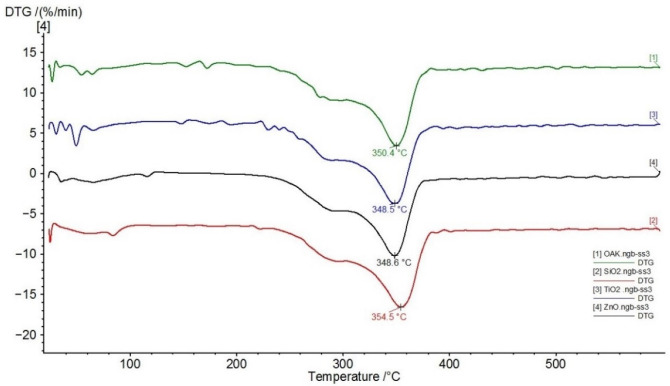
Differential thermal gravimetry (DTG) curves of the samples treated with 3% dispersion of nanoparticles (1—Oak wood, green; 2—SiO_2_, red; 3—TiO_2_, blue; 4—ZnO, black).

Thermal analysis proved that the weight loss (5%, TG curve, not shown) of unmodified oak wood starts at temperatures below 100 °C (max. at 54 °C, DTG curve) due to the removal of absorbed water. The second weight-loss step was detected at a temperature of 290 °C (shoulder on TG curve). The most significant wood decomposition was observed at 350 °C (Figure 1).

**Figure 2:**
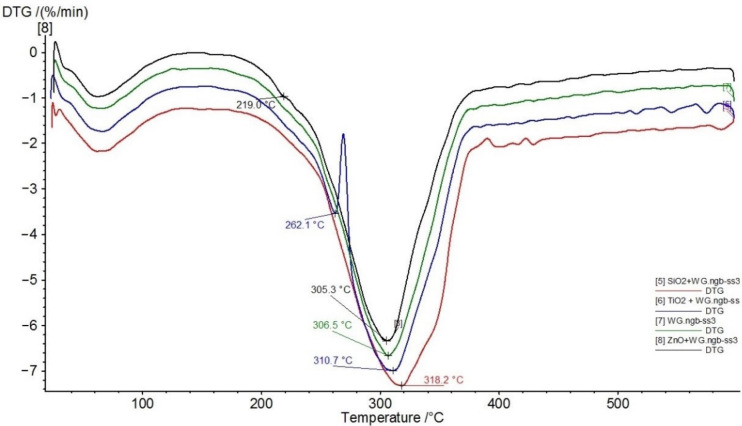
Comparison of DTG curves of the samples treated with 3% dispersion of nanoparticles in 20% aqueous solution of water glass (WG) (5—SiO_2_ + WG, red; 6—TiO_2_ + WG, blue; 7—WG, green; 8—ZnO + WG, black).

According to Sebio-Puñal et al. [27], the peak at 350 °C can be easily assigned to cellulose and the shoulder at 290 °C can be assigned to holocellulose. The weight loss with a maximum decrease at 290 °C is in good agreement with the non-glucosic saccharide content in oak wood [28]; therefore, this peak can be attributed primarily to hemicellulose decomposition. The decompositions of hemicellulose, cellulose, and lignin take place in a relatively narrow range of temperatures, partially overlapping. The complex structure of lignin leads to degradation in a wide temperature range, which overlaps those of hemicellulose and cellulose; specifically, oak lignin degrades over a broad temperature scale [27,29,30]. Whole wood starts to thermally degrade at about 250 °C, caused by various reactions (dehydration, decarboxylation, decarbonylation, depolymerization, etc.) to produce carbon dioxide, carbon monoxide, water acetaldehyde, propenal, methanol, acetic acid, other volatile compounds, and tar and char residues [21,31]. To improve the flammability properties of oak wood, the environmentally friendly modifier sodium silicate (water glass) in combination with various nanoparticles were used. Figure 2 presents the comparison of the main decomposition peaks of the samples treated with 3% dispersion of nanoparticles in a 20% aqueous solution of WG.

Figure 1 and Figure 2 show that nanoparticles have only negligible effects, and the changes in thermal behaviour were influenced mainly by WG (Table 1). A similar observation was reported by Garskaite et al. [21] in Scots pine wood treated with sodium silicate and TiO_2_ nanoparticles. The dominant effect of WG was observed in the main weight-loss step. The drop in the temperature decomposition was approx. 36–44 °C compared with samples treated without WG, with a non-significant impact of the nanoparticles (Figure 1 and Figure 2, Table 1). Furthermore, the residues at a temperature of 600 °C were higher by approx. 8–12% for the samples treated with WG solution + nanoparticles compared with those treated with water + nanoparticles (Table 1). Alkali solutions strongly change the structures of wood components and accelerate their decomposition at elevated temperatures [32,33]. Thermogravimetry (TG) coupled with the Fourier transform infrared spectroscopy (FTIR) showed that the presence of WG increases the ratio of CO_2_ band to the carboxylic (C=O) band. The reduced intensities of the carboxylic groups in the treated wood spectra indicated possible extraction of the aliphatic compound during the alkali treatment. Some chemical reactions between TiO_2_ and the amorphous Na-O-Si gel upon heating were observed [21]. More intensive decomposition of wood treated with WG, and nanoparticles leads to a faster release of non-combustible gases, which slows down the combustion process. From this point of view, ZnO has the greatest influence among the nanoparticles investigated. This effect was also indicated by a reduction in enthalpy (Table 2). The impact of sodium silicate and various compounds for flame retardancy was investigated to find low-cost and less toxic flame retardants. Their retarding effect was evaluated [21,34,35,36] also by measuring the limiting oxygen index (LOI), and a good correlation between thermal analyses and LOI values was found.

### 3.2. Infrared Spectroscopy

**Figure 3:**
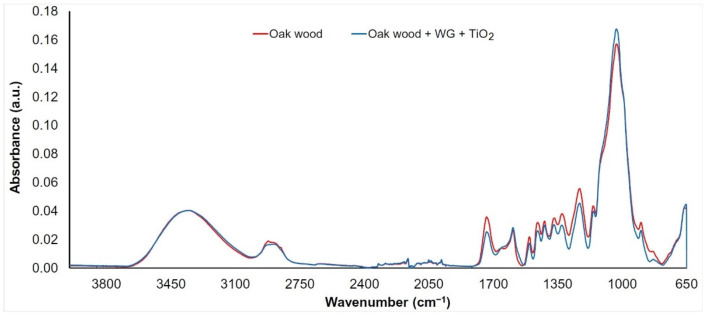
FTIR spectrum of oak wood coated with water glass and TiO_2_ nanoparticles.

**Figure 4:**
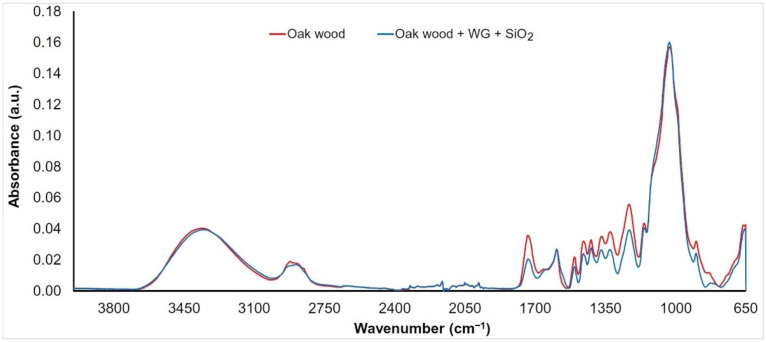
FTIR spectrum of oak wood coated with water glass and SiO_2_ nanoparticles.

**Figure 5:**
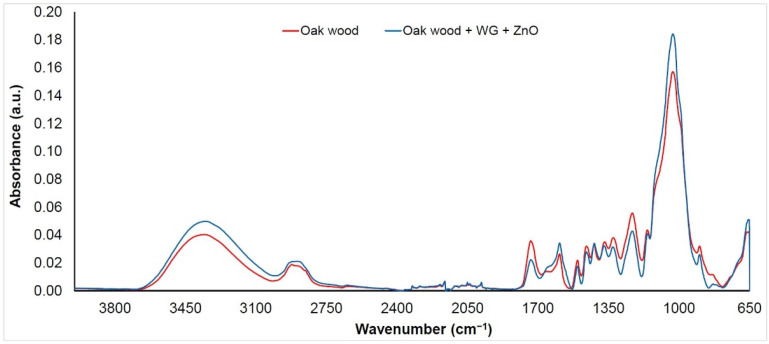
FTIR spectrum of oak wood coated with water glass and ZnO nanoparticles.

The FTIR spectra were measured on the wood surface before (denoted as “Oak wood”) and after the application of an aqueous solution of sodium silicate (WG) mixed with three types of nanoparticles (TiO_2_, SiO_2_, and ZnO, denoted as “Oak wood + WG + nanoparticles”). Given that the changes in the spectra were almost the same for each of the mixtures used, we decided to evaluate them together. As can be seen (Figure 3, Figure 4 and Figure 5), band changes in the range of 3100 to 3600 cm^−1^ are negligible. This wide band is assigned to O−H vibrations in cellulose, hemicellulose, and lignin structures [37]. An exception is the surface treated with an aqueous solution of ZnO + WG. The growth in the band with a peak at 3380 cm^−1^ can, in this case, be influenced by the presence of a wide band characteristic of ZnO, as well as the possible presence of water molecules absorbed on the surface of ZnO nanocrystalline powder [21,38]. The interval between 2950 and 2850 cm^−1^ (symmetric and asymmetric C−H stretching vibrations in aliphatic compounds) [39] shows similar behaviors. In the area from the 1800 to 800 cm^−1^ bands assigned to stretching and deformation vibrations of all wood components (fingerprint region), more significant changes in absorbances were recorded.

A decrease in band absorbance on 1735 cm^−1^ (C=O stretching in unconjugated carbonyl groups) of more than 35% (Figure 3, Figure 4 and Figure 5) was observed on the treated samples, indicating changes in several functional groups in lignin and hemicelluloses (carbonyls, aldehydes, ketones, and carboxylic acids) [40,41]. This decrease may be due to changes in the polysaccharides due to the action of an alkaline Na_2_SiO_3_ solution. Alkaline treatment of wood promotes the deacetylation of hemicelluloses and has an effect on the degradation of xylans [42]. A separation of xylans and glucomannans is also found in wood [21]. The band around 1600 cm^−1^ (C=C stretching conjugated with an aromatic ring in lignin) is practically unchanged. An exception is a sample treated with a solution containing ZnO nanoparticles, where an increase in absorbance of about 25% was observed. This may be due to overlap with the relatively wide band present in the ZnO spectrum (Zn−O stretching vibrations in crystalic structures) [43,44]. The band absorbance near 1500 cm^−1^ (C=C stretching conjugated with aromatic ring in lignin) decreased by around 20%. This mainly indicates a decrease in the number of methoxyl groups, confirming the decrease in lignin content [45,46].

The bands also decreased at 1460 cm^−1^ (asymmetric CH_3_ bending in methoxyl groups in lignin), 1370 cm^−1^ (symmetric and asymmetric CH_3_ bending), 1320 cm^−1^ (C−O vibration in syringyl derivatives), and 1235 cm^−1^ (C−O stretching vibration in xylan and syringyl ring), which are associated with lignin and hemicelluloses [47,48,49,50]. Their decrease supports the assumption that lignin degradation is caused by the presence of an alkaline environment (the pH of the water glass solution used in the experiment was more than 10). In contrast to previous trends, a slight increase in the 1030 cm^−1^ band (C−O deformation vibrations in cellulose) was observed (Figure 3, Figure 4, and Figure 5). Since this band does not occur in the spectra of the nanoparticles used, the increase is probably supported by a partial overlap and superposition with the band characteristic of water glass (Si−O stretching vibrations) [51]. The band at 897 cm^−1^ is associated with C−O−C stretching vibrations at glycosidic linkage in cellulose [52]. Absorbance on this band shows a permanent decrease, which confirms the degradation of cellulose.

### 3.3. Scanning Electron Microscopy–X-ray Spectroscopy Observations

**Figure 6:**
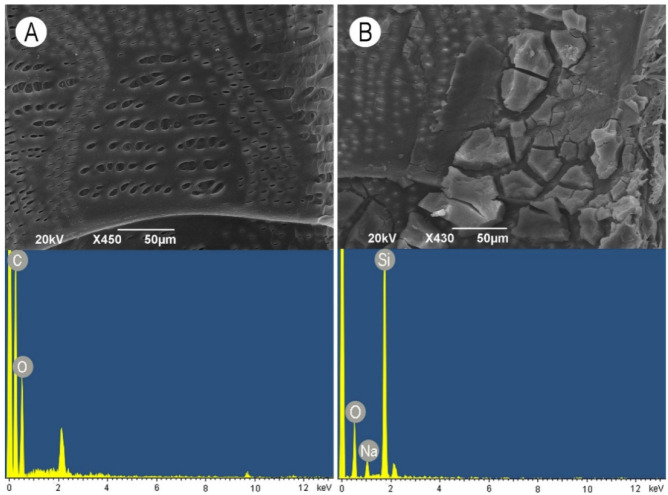
Scanning electron microscopy images of earlywood vessel wall surfaces (upper images) accompanied with the representative EDX spectra showing the elemental composition (bottom images). (**A**) Untreated control oak wood, radial section, scale bar = 50 μm. (**B**) Treatment with the aqueous solution of water glass, tangential section, scale bar = 50 μm.

In untreated control wood samples, the vessel walls were found to be free of any deposits and cell wall debris. The vessel-ray pits showed both large and small apertures (Figure 6A). On the contrary, massive layers were observed on vessel wall surfaces when wood was treated with the WG solution. Non-continuous, deeply cracked deposits containing sodium silicate partially covered the cell wall surfaces and pit apertures (Figure 6B).

**Figure 7:**
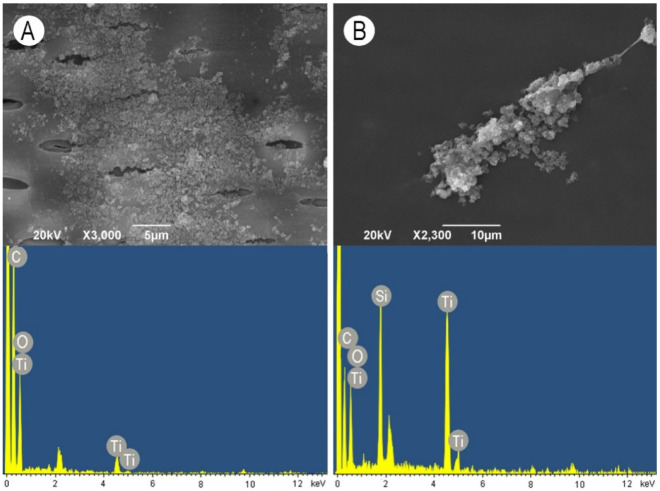
Scanning electron microscopy images of earlywood vessel wall surfaces (upper images) accompanied with the representative EDX spectra showing the elemental composition (bottom images). (**A**) TiO_2_ nanoparticles, radial section, scale bar = 5 μm. (**B**) Aqueous solution of water glass in combination with TiO_2_ nanoparticles, radial section, scale bar = 10 μm.

When TiO_2_ nanoparticles were applied alone, they formed very thin, non-continuous layers of apparently small particles. The particles penetrated into the pits and were deposited on the pit borders and inside the pit apertures (Figure 7A). The addition of WG in combination with TiO_2_ nanoparticles caused the formation of extremely large aggregates attached to the vessel wall surface (Figure 7B).

**Figure 8:**
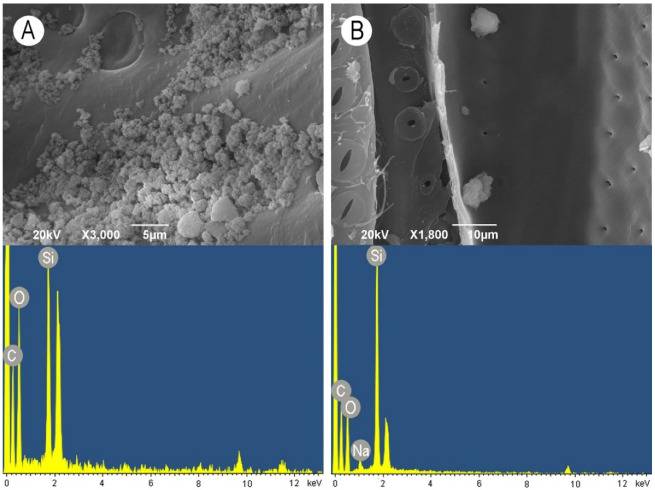
Scanning electron microscopy images of earlywood vessel wall surfaces (upper images) accompanied with the representative EDX spectra showing the elemental composition (bottom images). (**A**) SiO_2_ nanoparticles, radial section, scale bar = 5 μm. (**B**) Aqueous solution of water glass in combination with SiO_2_ nanoparticles, radial section, scale bar = 10 μm.

**Figure 9:**
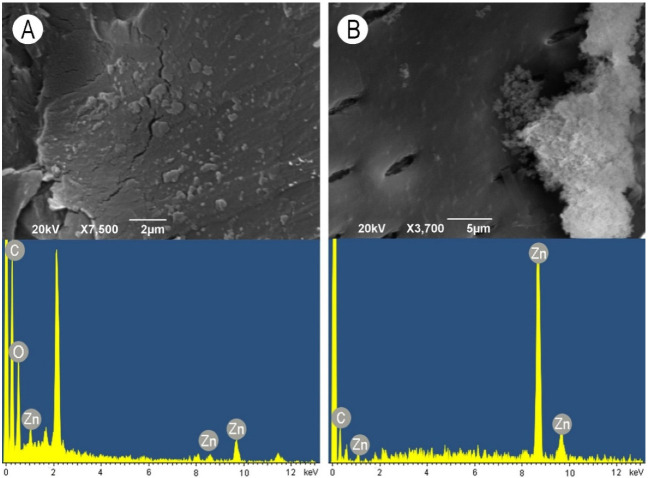
Scanning electron microscopy images of earlywood vessel wall surfaces (upper images) accompanied with the representative EDX spectra showing the elemental composition (bottom images). (**A**) ZnO nanoparticles, radial section, scale bar = 2 μm. (**B**) Aqueous solution of water glass in combination with ZnO nanoparticles, radial section, scale bar = 5 μm.

SiO_2_ nanoparticles did not form a continuous layer but rather clumps consisting of relatively larger particles. Smaller particles again penetrated into the pit apertures, but due to the large size of some clumps, some pits were fully covered with SiO_2_ nanoparticles (Figure 8A). When the WG solution was applied in combination with SiO_2_ nanoparticles, mostly roundish aggregate deposits were observed (Figure 8B). ZnO nanoparticles were deposited on the vessel wall surface as a relatively thin continuous layer. Except for some small cracks, a smooth layer covered the cell wall surface and pits (Figure 9A). On the contrary, the addition of WG in combination with ZnO nanoparticles caused the formation of very large aggregates consisting of small nanoparticles covering the surface of vessel-ray pits (Figure 9B). The elemental composition of both the examined nanoparticles and the WG solution was confirmed by the EDX analysis, as shown in the images at the bottom of Figure 6, Figure 7, Figure 8 and Figure 9.

## 4. Conclusions

Three types of nanoparticles (TiO_2_, SiO_2_, and ZnO) and sodium silicate were investigated to improve the fire resistance of oak wood. Thermal analyses show the most considerable wood decomposition at a temperature of 350 °C with a non-significant influence of the nanoparticles. On the other hand, the presence of sodium silicate resulted in a rapid drop in the decomposition temperature. Faster decomposition of wood treated with sodium silicate and nanoparticles led to a faster release of non-combustible gases, which slowed down the combustion process. From this point of view, ZnO has the greatest influence among the nanoparticles investigated. This effect was also indicated by a reduction in enthalpy. The results demonstrated that wood modifications using sodium silicate and nanoparticle systems have potentially enhanced flame retardant properties.

## Figures and Tables

**Table 1:** Thermal degradation temperatures and residue at 600 °C from TG and DTG analyses.

Sample	*T*1 (°C)	*T*2 (°C)	Residue at 600 °C (%)
Oak wood	290.0	350.4	18.3
Oak wood + WG	–	306.5	25.8
Oak wood + TiO_2_	294.0	348.5	17.5
Oak wood + WG + TiO_2_	262.1	310.7	26.0
Oak wood + SiO_2_	294.0	354.5	16.2
Oak wood + WG + SiO_2_	–	318.2	25.3
Oak wood + ZnO	293.0	348.6	17.2
Oak wood + WG + ZnO	219.0	305.3	28.4

**Table 2:** Peak temperatures and enthalpy obtained from DSC curves.

Sample	*T*1 (°C)	Δ*H* (J/g)	*T*2 (°C)	Δ*H* (J/g)
Oak wood	352.5	60.1	440.3	57.9
Oak wood + WG	–	–	406.9	13.7
Oak wood + TiO_2_	351.5	59.6	450.8	78.1
Oak wood + WG + TiO_2_	364.0	7.8	–	–
Oak wood + SiO_2_	358.4	53.1	409.4	59.8
Oak wood + WG + SiO_2_	374.7	7.1	442.6	10.3
Oak wood + ZnO	351.1	69.2	434.5	52.7
Oak wood + WG + ZnO	357.6	–1.3	–	–

## Data Availability

The data are available within the article.
